# Carbon Nanotubes Substrates Alleviate Pro-Calcific Evolution in Porcine Valve Interstitial Cells

**DOI:** 10.3390/nano11102724

**Published:** 2021-10-15

**Authors:** Luisa Severino Ulloa, Fabio Perissinotto, Ilaria Rago, Andrea Goldoni, Rosaria Santoro, Maurizio Pesce, Loredana Casalis, Denis Scaini

**Affiliations:** 1 Dipartimento di Fisica, Università di Trieste, Piazzale Europa 1, 34127 Trieste, Italy; luisa.ulloaseverino@unityhealth.to (L.S.U.); fabio.perissinotto@inserm.fr (F.P.); ilaria.rago@uniroma1.it (I.R.); 2 Elettra-Sincrotrone Trieste S.C.p.A., Basovizza, 34149 Trieste, Italy; andrea.goldoni@elettra.eu; 3 Unità di Ingegneria Tissutale Cardiovascolare, Centro Cardiologico Monzino, IRCCS, 20138 Milan, Italy; rosaria.santoro@cardiologicomonzino.it (R.S.); maurizio.pesce@cardiologicomonzino.it (M.P.); 4 Area di Neuroscienze, Scuola Internazionale Superiore di Studi Avanzati, Via Bonomea 265, 34136 Trieste, Italy; 5 Faculty of Medicine, Imperial College London, London W12 0NN, UK

**Keywords:** carbon nanotubes, chemical vapor deposition, nanomorphology, valve interstitial cells, cell membrane

## Abstract

The quest for surfaces able to interface cells and modulate their functionality has raised, in recent years, the development of biomaterials endowed with nanocues capable of mimicking the natural extracellular matrix (ECM), especially for tissue regeneration purposes. In this context, carbon nanotubes (CNTs) are optimal candidates, showing dimensions and a morphology comparable to fibril ECM constituents. Moreover, when immobilized onto surfaces, they demonstrated outstanding cytocompatibility and ease of chemical modification with ad hoc functionalities. In this study, we interface porcine aortic valve interstitial cells (pVICs) to multi-walled carbon nanotube (MWNT) carpets, investigating the impact of surface nano-morphology on cell properties. The results obtained indicate that CNTs significantly affect cell behavior in terms of cell morphology, cytoskeleton organization, and mechanical properties. We discovered that CNT carpets appear to maintain interfaced pVICs in a sort of “quiescent state”, hampering cell activation into a myofibroblasts-like phenotype morphology, a cellular evolution prodromal to Calcific Aortic Valve Disease (CAVD) and characterized by valve interstitial tissue stiffening. We found that this phenomenon is linked to CNTs’ ability to alter cell tensional homeostasis, interacting with cell plasma membranes, stabilizing focal adhesions and enabling a better strain distribution within cells. Our discovery contributes to shedding new light on the ECM contribution in modulating cell behavior and will open the door to new criteria for designing nanostructured scaffolds to drive cell functionality for tissue engineering applications.

## 1. Introduction

Among all aortic valve diseases widely diffuse in the Western world, Calcific Aortic Valve Disease (CAVD) is the main cause of aortic stenosis and represents a major healthcare burden [1]. In adults, the aortic valve (AOV) is predominately composed of two types of cells: valve endothelial cells (VECs) and valve interstitial cells (VICs). CAVD is a sclerotic process that goes together with a phenotypic modification of VICs. In healthy aortic valves, VICs are a heterogeneous population constituted mostly of smooth muscle cells and fibroblasts, with a small percentage of myofibroblasts (about 5%) [2,3,4]. It has been shown that in the CAVD pathological condition, the fraction of myofibroblasts rises within the overall cellular population (up to 30%) [4,5,6]. The disease is also associated with changes in the composition, organization, and mechanical properties of the extracellular matrix (ECM)-embedding VICs. ECM alterations are not simply the result of dysfunction of valve cells. They seem, in turn, to contribute to the progression of the pathology, impairing cellular homeostasis and altering molecular signaling [7], determining a phenotypic differentiation of myofibroblasts into osteoblast-like cells, ultimately responsible for the formation of calcium nodules [8]. Therefore, given this body of evidence, it is crucial to clarify the role of the cell/ECM interaction in promoting mechanical heterogeneity of the valve tissue and, ultimately, cellular sensibility to pro-pathological molecular or genetic cues [9]. It has been demonstrated that a stiff substrate causes an increase in the elastic modulus of VICs plated atop, leading to a significant rise in the loading of α-smooth muscle actin (α-SMA) in stress fibers, an indicator of myofibroblast maturation [10,11]. Moreover, it has been shown that in the onset of CAVD, the ordered organization of collagen fibers, typical of healthy AOV, is lost, and local variations in ECM mechanics arise [12,13]. On the other hand, other factors might critically impact VICs’ phenotype evolution, such as the ECM (nano)morphology, sometimes associated with unregulated stress relaxation and excessive trans-valvular pressure [14,15].

In order to sort out the impact of specific (nano)topography and physicochemical properties on the behavior of VIC-interfaced cells [16,17], we searched for artificial ECM-mimicking biomaterials from tissue engineering [18]. In particular, we have focused on carbon nanotubes (CNTs), showing dimensions and morphology comparable to natural fibrillar ECM constituents [19]. CNTs are nanomaterials characterized by in vitro and in vivo stability and cytocompatibility when immobilized onto surfaces [20,21,22,23,24], outstanding electrical conductivity, and ease of chemical functionalization to endow them with ad hoc functionalities [25,26].

In the present study, we interfaced porcine valve interstitial cells (pVICs) to pristine multi-walled carbon nanotubes (MWNTs) carpets, comparing their properties to those of cells plated on glass controls in standard culture medium, a condition known to induce pro-pathological endorsement of pVIC cells [27]. With this study, we aimed to disclose the impact of CNT nanomorphology on pVIC characteristics, attempting a discrete cell analysis. To pursue the investigation, we directly grew CNTs on supporting glass slides by catalytic chemical vapor deposition (CCVD) [24]. The optical transparency of our thin CNT films allows associating immunofluorescence imaging, revealing cell morphology, with Atomic Force Microscopy (AFM) force-spectroscopy experiments, highlighting cell stiffness.

## 2. Materials and Methods

### 2.1. CNTs Synthesis

Multi-walled carbon nanotubes were synthesized by thermal decomposition of acetylene (carbon source) over a thin catalytic layer of iron nanoparticles [23], using fused silica (SiO_2_) glass slides as supporting substrates. The procedure was developed internally in our laboratory and described elsewhere [24]. Briefly, samples were cleaned by the Radio Corporation of America (RCA) method, followed by the deposition of a thin layer of iron (1–2 nm in thickness) directly on the silica slides using electron beam (e-beam) evaporation. Iron film thickness was monitored with an in situ quartz crystal microbalance (STM-2, Scanwel, UK). Since the uniformity of the catalyst layer is crucial for CNT growth, an average deposition rate of 0.2 Å/sec was adopted. Subsequently, the as-evaporated substrates were placed in a high vacuum reaction chamber and annealed (4 min at 650–670 °C in an H_2_-reducing atmosphere) to remove iron oxides that were possibly formed. Furthermore, the annealing treatment broke down the continuous iron film into nanoparticles, acting as nucleation sites for CNTs growth. After this pre-treatment, the carbon source was introduced in the reaction chamber up to a partial pressure of about 10–20 mbar. The sample temperature was increased to 730 °C and reaction time was limited to 30 s, resulting in the formation of a uniform, optically transparent, carpet of CNTs. For the purposes of this work, we used samples characterized by a CNT film thickness in the range of 0.5–1.0 µm, ultimately able to guarantee the needed transparency together with a good surface coverage. After CNT synthesis, samples were allowed to cool down to room temperature and used as they were removed from the reaction chamber.

### 2.2. Isolation and Culture of Porcine Aortic Valve Interstitial Cells

Aortic valves of 6–9-month-old pigs were acquired from a local abattoir and porcine aortic VICs were aseptically isolated following the procedure described by Santoro R. et al. [28]. Briefly, a first incubation for 5 min on a shaker at 37 °C in Collagenase Type II (Worthington) solution (1000 U/mL) was performed to remove the endothelial layer. Subsequently, to isolate pVICs, a second incubation (2 h) was carried out under the same conditions. Cells were then seeded for in vitro amplification on plastic cell culture dishes (10 cm in diameter) coated with 1% gelatin and allowed to grow in Dulbecco’s Modified Eagle Medium (DMEM, Thermo Fisher Scientific, Waltham, MA, USA) containing 150 U/mL penicillin/streptomycin, 2 mM L-glutamine, and 10% fetal bovine serum (all reagents from Sigma-Aldrich, St. Louis, MO, USA). Cells were seeded onto glass and CNT-modified substrates at a density of about 10^5^ cells/cm^2^ followed by culturing for up to 72 h in the same culturing medium in the incubator (37 °C, 5% CO_2_). Before cell plating, substrates were cleaned for 1 h in pure ethanol and subsequently treated for 5 min with a low-power oxygen plasma. Samples were sterilized under the UV light of a biosafety hood for 20 min. Cells were plated on both substrates (glass control coverslips or CNT covered ones) without any further adhesion coating. After 12 or 72 h, cells were fixed in paraformaldehyde (PFA, 4% in phosphate-buffered saline solution, PBS) for subsequent immunostaining and AFM/SEM analysis.

### 2.3. Immunofluorescence Assay

Cellular samples interfaced to glass control coverslips and CNTs were washed 3 times with PBS and then fixed with 4% PFA for 30 min at room temperature (RT). After fixation, samples were permeabilized with a 0.5% Tween-20 PBS solution for 10 min and washed 3 times with 0.1% Tween-20 in PBS. After permeabilization, samples were blocked with PBS containing 5% fetal bovine serum (FBS) for 1 h. Samples were incubated for 30 min at RT with phalloidin directly coupled to an Alexa Fluor 594 fluorophore (ThermoFisher Scientific, Waltham, MA, USA) at 1:10 dilution in a blocking solution (5% FBS in PBS). After 3 washes of 5 min each with PBS, coverslips were incubated with an anti-vinculin antibody (mouse monoclonal, VIIF9 (7F9); Sigma-Aldrich, St. Louis, MO, USA) or an anti-α-SMA antibody (Monoclonal Mouse Anti-Human, Clone 1A4, Agilent Technologies, Santa Clara, CA, USA) for 2 h at RT at, respectively, 1:20 and 1:50 dilution in blocking solution (5% FBS in PBS). Samples, washed 3 times for 5 min with PBS, were subsequently incubated with the secondary antibody for 2 h at RT at the appropriate dilution in the same blocking solution. Two secondary antibodies were alternatively used, a goat anti-mouse IgG coupled to Alexa Fluor 594 at 1:500 dilution, or a goat anti-mouse IgG coupled to Alexa Fluor 488 at 1:500 dilution (both from ThermoFisher Scientific, Waltham, MA, USA). After 3 washes of 5 min with PBS, the nuclei are stained by incubation with DAPI (Sigma Aldrich, St. Louis, MO, USA) in PBS (1:3000) for 5 min.

pVIC membrane patches adherent to both substrates were highlighted, after cell lysis and fixation (see below), using 1,1′-Dioctadecyl-3,3,3′,3′-Tetramethylindocarbocyanine Perchlorate (DiI, ThermoFisher Scientific, Waltham, MA, USA), employed at 1:100 dilution for 15 min.

Samples were carefully rinsed two times in PBS and once in milliQ H_2_O, eventually sandwiched with a thin glass slide, and then visualized under an inverted fluorescence microscope (Eclipse TiU, Nikon Corporation, Tokyo, Japan). All image analyses were conducted using the open-source program Fiji version 2.1.0 [29] and the Wolfram Mathematica technical computation suite (version 12.1.0.0, Wolfram Research, Inc., Champaign, IL, USA).

### 2.4. pVICs Lysis to Expose the Surface-Interacting Plasma Membrane

Cells were plated on both substrates at a confluence of 5 × 10^5^ cells/cm^2^. Before cell seeding all the substrates were washed with ethanol and exposed to a plasma-cleaning treatment. After 72 h, cells were subjected to osmotic shock to reveal the underlying cell membrane exploiting a protocol previously described by Ziegler U. et al. [30]. Briefly, cells were washed with an isotonic ice-cold solution (20 mM PIPES, 150 mM KCl, pH 6.2), incubated with a hypotonic buffer (4 mM PIPES, 30 mM KCl, pH 6.2) for 3 min on ice, and flushed out using 5 mL of the same buffer through a 25-gauge needle. Subsequently, samples were left for 30 min in a high salt solution (2 M NaCl, 2.7 mM KCl, 1.5 mM KH_2_PO_4_, 1 mM Na_2_HPO_4_, pH 7.2) at room temperature. Finally, the obtained membrane patches immobilized on the two substrates were fixed in 4% PFA and washed 3 times in PBS for subsequent investigation.

### 2.5. Evaluation of α-SMA and Vinculin Expression

Cell expression of α-SMA and vinculin were evaluated for every cell and normalized on cell area by means of the phalloidin/actin signal. In the case of α-SMA, for both glass controls (11 fields from 3 independent experiments) and carbon nanotubes samples (9 fields from 3 independent experiments), the surface ratio of α-SMA on actin signals was computed (values are expressed in µm^2^/µm^2^). Focal adhesions (FAs) analysis was conducted by subtracting the cytosolic background signal and evaluating the area of vinculin-positive regions normalized on the total cell area for every categorized cell morphology present on glass controls (9 fields from 3 independent experiments), and on CNT samples (9 fields from 3 independent experiments). Values are expressed in terms of µm^2^/µm^2^.

### 2.6. Cell Density, Degree of Circularity and Branching Assay

Cells were plated on both substrates at a confluence of 1 × 10^5^ cells/cm^2^. After 12–72 h, cells were fixed and labelled according to the immunofluorescence protocols previously described. Phalloidin and 4′,6-diamidino-2-phenylindole (DAPI) were included in our protocols to estimate cell density and shape, the latter for classification and normalization purposes. Regarding the evaluation of cell densities for the two different substrates, at the 12 h time point, 75 fields were analyzed from 9 independent experiments for each condition, while at the 72 h time point, 125 fields from 12 independent experiments were considered for each condition. Cell density was computed as the ratio between the number of DAPI-highlighted nuclei and the total area of the imaged field of view, and expressed as 1/µm^2^.

The degree of circularity (DOC) of these cells was evaluated as the ratio between the radius of the circle with the same area of the cell and the radius of the circle with a circumference of the same length as the cell perimeter. It is a dimensionless parameter expressed by the formula:

$$DOC=\sqrt{\frac{A}{\pi }/\frac{P}{2\pi }}=\frac{2\sqrt{\pi A}}{P}$$

Cell branching was evaluated by manually counting the number of cusps for every cell inside a field of view (6 fields for every condition from 3 independent experiments). For example, while a circular cell has no cusps, drop-, spindle- and triangular-shaped ones have values of 1, 2, and 3, respectively.

### 2.7. Sorting of pVICs Morphologies When Grown on CNTs Substrates

We performed a discrimination and classification procedure of pVICs based on cell morphology. Cells were fixed and labelled with Phalloidin and DAPI after 12 and 72 h of growth according to the previously described immunofluorescence protocols. About 2500 cells for each condition were analyzed (evaluated on 25 fields from 3 independent experimental sessions) in terms of morphology, and associated, based on that, with one of the three main cellular constituents of pVIC cultures: smooth muscle cells (SMc), fibroblast (Fib), or myofibroblasts (myFib) [2].

Initially, a sub-population of cells (about 250 cells per condition from 12 h samples) was manually classified based on a qualitative cell shape analysis. Cells were grouped into three different classes: cells with small areas and a characteristic spindle-like shape that we associated with SMc [31,32,33,34], cells characterized by larger areas and a cuspate shape, associated with myFib [33,34,35,36,37], and cells presenting intermediate elongated shapes that we associated with Fib [32,33,34,36,37,38].

From this classification, we have noticed that SMc-associated morphologies were characterized by a ratio between their longer and shorter axes larger than 3; for Fib-associated ones, such a ratio assumes a value between 1.5 and 3.0, while myFib-associated have revealed a ratio between 1 and 1.5. From this evidence, we set up an automatic cell classifier based on the Wolfram Mathematica ClusterClassify function, evaluating the cell elongation value through the ComponentMeasurment function. Elongation was internally defined by the software as 1-width/length. As expected, the clustering procedure automatically highlighted three well-distinct classes, where low elongation (0 < x < 0.551) refers to myFib-associated geometries, intermediate elongation (0.551 < x < 0.817) refers to Fib-associated, and high elongation (0.817 < x < 1) refers to SMc-associated. We tested the quality of the obtained classifier function (KMedoids) on the initial, manually sorted, sub-population (777 cells from both substrates), and we obtained an accuracy of 0.968 ± 0.022 (in Figure S2 some representative outcomes from the automatic cell clustering process).

The procedure allowed for accurate quantitative and automatic classification of every cell based on its morphology at both 12- and 72 h time points. We considered only cells falling within one of the three classes with a probability higher than 90%, otherwise they were not included in the analysis. Less than 5% of cells were excluded. This value is smaller than the error in cell classification we usually found; consequently, we can state it is not impacting our experimental outcomes.

### 2.8. CNTs and Cell Characterization by Field Emission Scanning Electron Microscopy

Scanning Electron Microscopy (SEM) imaging was performed on a Gemini SUPRA 40 SEM (Carl Zeiss NTS GmbH, Oberkochen, Germany). CNT carpets were imaged as produced at an acceleration voltage of about 6 keV.

Prior to SEM visualization of cell-interfaced glass and CNT samples, cells were washed with 0.1 M cacodylate buffer (pH 7.2) and subsequently fixed for 1 h at RT with 2% glutaraldehyde (Fluka Chemie GmbH, Buchs, Germany) in a 0.1 M cacodylate buffered solution. Cultures were carefully rinsed with cacodylate buffer and dehydrated by soaking them in a sequence of water/ethanol solutions at progressively higher alcoholic concentrations (30%, 50%, 70%, 80%, 90%, 95%, and 100% ethanol) for 10 min each. Samples were then left to dry at 4 °C overnight. In order to avoid charge accumulation during SEM analysis, all samples were Au metallized in a metal sputter coater (Polaron SC7620, Quantum Design GmbH, Darmstadt, Germany).

SEM characterization of membrane patches after cell lysis were performed on glass substrates after the deposition of a thin layer of gold. Membranes adherent to CNT carpets were instead visualized without metallization by the fact they were already sufficiently conductive. Images were acquired operating at very low acceleration voltages to avoid charge accumulation (0.9–1.5 keV).

### 2.9. Atomic Force Microscopy Imaging and Force Spectroscopy

Atomic Force Microscopy was used to evaluate the thickness, morphology and roughness of surface-interacting cell membrane patches on flat glass controls and on CNT carpets. AFM images were acquired using a commercial AFM (NT-MDT Solver Pro, NT-MDT, Moscow, Russia) mounted on an inverted fluorescence microscope (Eclipse TiU, Nikon Co., Tokyo, Japan). All AFM measurements were carried out at room temperature working in dynamic mode. Cell membrane fragments were stained for 15 min with DiI diluted in deionized water (mQ, 1:100) to make membrane patches visible and allow precise AFM tip positioning. After 3 additional washes in H_2_O mQ, samples were left to dry at room temperature. The samples were imaged using cantilevers characterized by about 0.6 nN/nm in force constant and 65 kHz in resonance frequency (HQ:NSC36/C from MikroMasch Co., Tallinn, Estonia). Images of 512 × 512 pixel^2^ were acquired at a 0.3 lines/second scan speed. The open-source scanning probe images analysis software Gwyddion [39] was used to analyze all AFM images (version 2.56). Root mean square line roughness (R_rms_) was defined internally to the software as the average of the measured height deviations taken within the evaluation length and measured from the mean line of the selected profile (ASME B46.1-1995, ISO 4287-1997, ISO 4287/1-1997).

The cells’ height and stiffness were evaluated by means of AFM imaging and force spectroscopy, respectively. For this purpose, cells were seeded on both substrates at a confluence of 2 × 10^5^ cells/cm^2^. Before seeding, all the substrates were washed with pure ethanol and plasma cleaned (see Section 2.2 for details). After 72 h of culturing, cells were fixed in 4% PFA, and their nuclei were made visible by staining them with DAPI (Sigma Aldrich, 1:3000 in PBS) for 5 min. Before force spectroscopy measurements, samples were carefully washed with PBS. AFM measurements were carried out at room temperature working in contact mode in liquid environment (PBS). Stiffness assessment was conducted, taking advantage of the force spectroscopy capabilities integrated in the NT-MDT control and analysis software (Nova-Px 3.4, NT-MDT Co., Moscow, Russia). Briefly, during force spectroscopy the displacement of a calibrated AFM cantilever is measured while it is moved against a surface. The data are subsequently converted into a force/indentation curve through the knowledge of the cantilever spring constant and AFM optical lever. Indentation was conducted by placing the cantilever tip above the cell nucleus (made fluorescent by DAPI staining). This strategy enabled high measurement reproducibility and semi-automatic probe placement. Tipless cantilevers with a nominal elastic constant of about 0.03 nN/nm and a resonance frequency of about 10 kHz (CSG11-B/tipless, NT-MDT Co., Moscow, Russia) were employed. An 8.0 ± 0.4 µm diameter borosilicate glass bead (No. 9008, Duke Standards^TM^, Fremont, CA, USA) was glued at the end of the cantilever using a UV-curable adhesive (Norland Optical Adhesive 61, Norland Products, Inc., East Windsor, NJ, USA). Before use, all the cantilevers were characterized in terms of their effective elastic constant by means of the thermal method [40] integrated into the AFM software, and in terms of bead diameter by means of electron microscopy (Figure S3). Force spectroscopy measurements were performed at a constant speed (1.5 µm/s) and triggered to a maximum sample indentation of 250 nm (about 5% of the average maximum cell height, see Figure S4B). Such indentation value cleared the measurement out from substrate contribution, minimizing at the same time its susceptibility to cell nucleus stiffness. Compliance of the material under the tip was determined by fitting the data with the Hertzian model for a spherical indenter [41]. For each condition, about 80 cells from 3 independent experiments were measured and cell stiffness was described in terms of Young’s Modulus (E) and expressed in kPa. Despite the fact that fixation, a procedure necessary to perform immunohistochemistry, alters the absolute value of stiffness for a specific cell, the relative variations between different experimental conditions of the same cell are generally maintained [42].

### 2.10. Statistical Analysis

For each experimental condition, at least 3 biological replicates were performed using different pools of cells. All statistical analysis was performed using the open-source R program version 4.1.1 [43], and the Wolfram Mathematica suite (version 12.1.0.0, Wolfram Research, Inc., Champaign, IL, USA). Data distribution was evaluated by Shapiro-Wilk test of normality and, based on the result, a bar chart or a box plot was chosen to graphically represent the data. Bar charts show mean ± standard deviation (SD). Box plots are plotted as median with boxes spanning from the 25th (1st quartile, Q1) to the 75th (3rd quartile, Q3) percentiles, with whiskers representing the 5th and 95th percentiles. Statistics between two independent samples were performed with t-test (normal distribution) or Mann-Whitney U test (non-normal distribution). Statistical differences between the three cell morphologies (SMc, Fib, and myFib) grown on the two different substrates (glass and CNTs) were evaluated through a two-way ANOVA followed by a Bonferroni post-test. For the sake of clarity, descriptive statistics used in the main text, if not otherwise stated, always refer to mean ± SD. Statistical significance was determined at *p* < 0.05, unless otherwise indicated. Significance was graphically indicated as follows: * *p* < 0.05, ** *p* < 0.01, *** *p* < 0.001.

## 3. Results

### 3.1. CNTs Fabrication and Characterization

**Figure 1:**
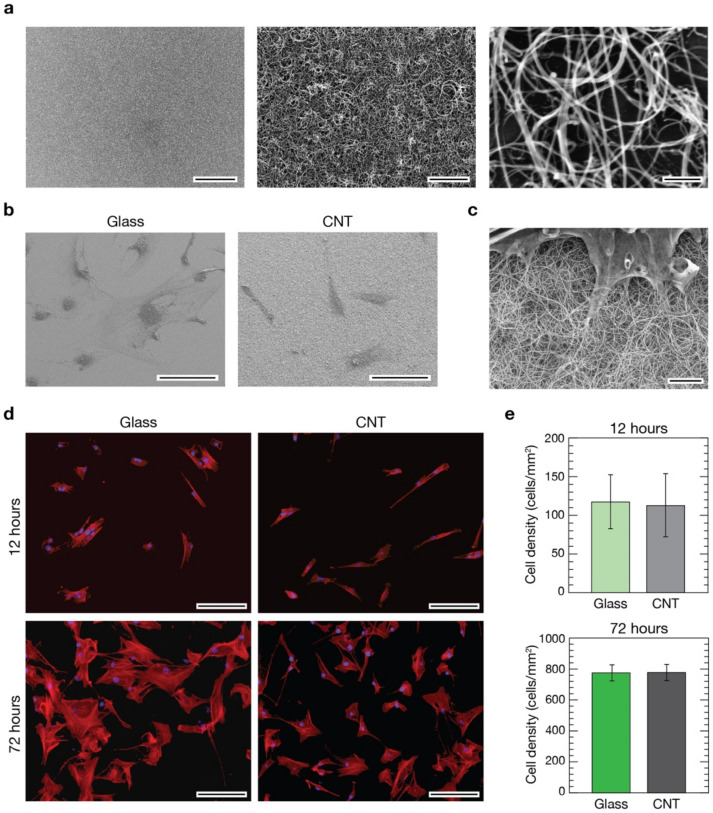
Evaluation of pVICs when developed on glass control and CNT substrates. (**a**) Scanning electron micrographs of CNT substrates at different magnifications pointing out carpet uniformity (left image) and CNT morphology (middle and right images). Scale bars: 50 µm, 2 µm, and 200 nm, respectively. (**b**) Low magnification electron micrographs of pVICs grown on a glass control coverslip (left) and a CNT substrate (right). Scale bars: 100 µm. (**c**) High magnification electron micrograph of a valve interstitial cell growth interfaced with a CNT-decorated substrate revealing the intimate contact between the cell and the filamentous nanotubes. Scale bar: 2 µm. (**d**) Immunofluorescence images of cells grown on glass (left column) and CNTs (right column) after 12 h (top row) and 72 h (bottom row) from seeding. Cell nuclei were stained in blue using DAPI, while cytoskeletal actin was pointed out in red using phalloidin. Scale bars: 100 µm. In (**e**), histograms comparing the cell densities calculated from the two different substrates at 12 (top) and 72 h (bottom) time points are shown. No differences were visible in terms of cell densities between controls and CNTs after 12 h (light green and light grey bars, respectively), nor after 72 h (dark green and dark grey bars, respectively, *t*-test, *p* > 0.05 at both time points).

Transparent CNT substrates were synthesized via CCVD directly on fused silica substrates (Figure 1) following a procedure recently developed in our laboratory and described elsewhere [23,24].

In short, in CCVD synthesis of CNTs, catalytic nanoparticles of well-defined size and density were deposited on the supporting surface and used as starting sites for the subsequent thermal growth of CNTs [23]. Using acetylene as a gaseous carbon precursor and setting the opportune synthesis parameters (i.e., substrate temperature, flow rate and reaction time), it is possible to cover the supporting substrate with a uniform continuous layer of entangled carbon nanotubes (Figure 1a). The thickness of the resulting CNT carpet can be easily controlled by the reaction time and partial pressure of acetylene in the reaction chamber [23]. If carpet height is lower than 1 µm, substrates are characterized by a good transparency to the visible light, making them functional for transmission optical microscopy [24]. For our purposes, we used fused silica substrates covered with a uniform layer of CNTs 0.5–1 µm in thickness (Figure S1A,B). High-resolution SEM imaging on CNT mats (Figure 1a, right) revealed a random, “spaghetti-like”, morphology with nanotube diameters in the range of 34 ± 9 nm. On these substrates, cells developed successfully (Figure 1b, right), and a tight interaction took place between CNTs and cells (Figure 1c). Interestingly, the morphology of CNT carpets and nanotubes dimensionality closely resemble the fibrous aspect of the extracellular matrix [23,44], making them an ideal substrate to interface living cells. Cell protrusions, such as filopodia and lamellipodia, extended above these nanostructured substrates perfectly integrate with them, making it difficult to distinguish where the CNT phase ends and the organic phase starts (Figure 1c and Figure S1C right).

### 3.2. Evaluation of pVICs Adhesion and Density on CNTs

In the CNT-decorated glass surfaces used within this research, nanotubes are firmly attached to the underneath glass support and, as already demonstrated in our previous works, in this condition they do not show any adverse effect on the viability of interfaced primary cells [20,21,22,23,24,45]. Despite these pieces of evidence, preliminarily to any further experiment, we have evaluated and compared cell densities in cultures developed above CNT substrates and control glass coverslips. Porcine aortic VICs were seeded onto CNTs and control substrates at an initial density of 10^5^ cells/cm^2^ followed by culturing for 12 or 72 h before immunofluorescence staining with 4′,6-diamidino-2-phenylindole (DAPI, to stain all cell nuclei) and Phalloidin (to stain actin filaments, see Section 2). Fluorescence analysis demonstrated that, after 12 h in culture, cells successfully developed on both substrates and the resulting cultures are homogeneous in their cellular distribution and densities (Figure 1d, top row). As expected, after 72 h the density of cells increased on control glass substrates and on CNT cultures while preserving a good coverage homogeneity (Figure 1d, bottom row). We evaluated the cellular densities for both substrates at the two time points (Figure 1e), and no significant differences were visible in cultures after 12 h from seeding on both controls and CNTs (119 ± 36 cells/mm^2^ and 114 ± 41 cells/mm^2^, respectively, on a total of about 1500 cells analyzed from 75 fields per condition from 9 independent experimental sessions) as well after 72 h (773 ± 48 cells/mm^2^ and 775 ± 52 cells/mm^2^ for controls and CNTs, respectively, on a total of about 15,000 cells on 125 fields from 12 sessions).

pVICs adhered, spread and proliferated similarly on both substrates. The observed homogeneity in cell distribution across the samples’ surfaces indicates similar cell motility in both conditions. These results confirmed once more the excellent cytological compatibility of surface-immobilized CNTs and, importantly, highlight their soundness as adhesion substrates for porcine VICs (Figure 1c and Figure S2).

### 3.3. CNTs Influence on VIC Morphology

**Figure 2:**
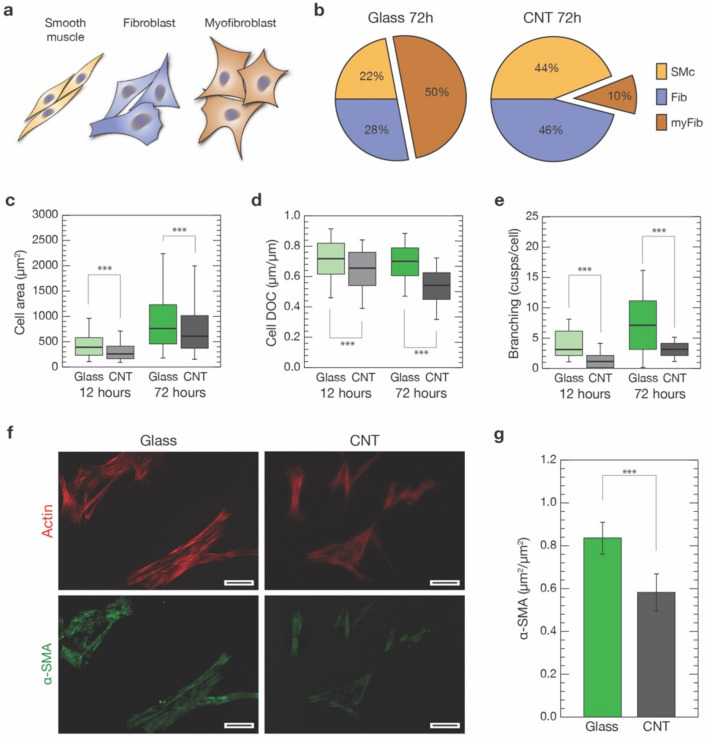
Morphological characterization of VICs grown on control glass and CNTs substrates. (**a**) Based on cell shape and elongation, we identified three distinct cell morphologies within our cultures: a spindle-like shape, associated with smooth muscles cells (SMc, orange), intermediate geometries ranging from unciform to sagittate shapes, associated with fibroblasts (Fib, blue), and large and cuspated shapes, associated with myofibroblasts (myFib, brown). (**b**) Percentages of the three different cell morphologies were evaluated in cultures developed for 72 h on both glass and CNTs. (**c**) Box plot pointing out cell dimensions after 12 h from seeding on glass controls (light green) and CNTs (light grey), and after 72 h (dark green and grey, respectively). (**d**) Degree of circularity (DOC) of cells developed on glass controls and CNTs for 12 and 72 h. (**e**) Number of cusps characterizing cells grown on glass controls and CNTs after 12 and 72 h. (**f**) Two representative fields of α-SMA staining (in green, bottom row) of cells developed for 72 h on glass and CNTs (actin filaments stained in red, top row). The generally lower and grainer fluorescence signal characterizing CNT samples should be noted. Scale bars: 50 µm. (**g**) Box plot summarizing α-SMA expression, normalized by the actin content, of cells developed interfaced for 72 h to glass substrates (dark green) and CNTs (dark grey). *** *p* < 0.001.

Once it had been demonstrated that the densities of the two cultures were equivalent on both substrates at the two different time points, we tried to disclose possible differences in cell characteristics induced by CNTs substrates (Figure 2). Based on the literature, our pVIC cellular population was mainly constituted by three different cell phenotypes: smooth muscles cells (SMc), myofibroblasts (myFib), and fibroblasts (Fib) [2].

An effective discrimination between these three cell phenotypes, and in particular between the former two, both positive to α-SMA signature, necessitates a combination of cell markers and mRNA/protein expression assays [3,46,47,48,49,50,51,52]. Moreover, being a post-process analysis, it is challenging to perform simultaneously with other investigations, making it at odds with the aim of our study. Instead, we were interested in univocally associating specific cell properties—such as morphology, cytoskeletal organization and stiffness—to a clearly identified cell category. For this reason, we have opted to base our classification on a morphological base. Specifically, based on histological analysis, we identified the three distinct cell morphologies characterizing our cultures (based on about 2500 cells analyzed per condition; see Section 2): a markedly bipolar lenticular shape, usually identifying smooth muscle cells; a spread stellate shape, associated with myofibroblasts; and intermediate geometries that range from unciform to sagittate shapes, that we linked to fibroblasts, as sketched in Figure 2a and Figure S2. We trained a Machine Learning Classifier (MLC) to validate our classification and subsequently automatically perform unbiased cell sorting of our cell population into one of these three morphological classes (see Section 2 and Figure S2). This process allowed us to univocally associate every experimental outcome from our single-cell analysis to a specific cell morphology. We tentatively associated such morphology-based subclasses to SMc, myFib or Fib cell types and performed further analysis.

Focusing our attention on the shape of the cells after 72 h from seeding (Figure 1d), our analysis revealed a significant change in the balance between these three cell morphologies over the two conditions (Figure 2b). After 72 h, half of the cell population on glass substrates was composed of SMc and Fib-associated cells (22% and 28%, respectively) and the remaining 50% was composed of the myFib ones (Figure 2b, left). On CNT-decorated substrates, the ratio between SMc and Fib was similar to cells grown on glass. However, they were now dominating (44% and 46%, respectively) with respect to cells exhibiting myFib morphology (10% of the total population, Figure 2b, right). In our experimental conditions, the amount of myofibroblasts observable on glass controls was, indeed, similar to the one characterizing unhealthy valves (e.g., affected by CAVD), while in the case of cells grown on CNTs, the amount of myofibroblasts was very similar to the healthy valve case [27,53]. In terms of cell dimensions, we observed a predictable increase in cell area after 72 h when compared to 12 h, on both substrates. Regardless, cells developed on CNTs always appeared to be significantly smaller in terms of the projected area when compared to controls (Figure 2c). In particular, when analyzing the cell degree of circularity (DOC, see Section 2) (Figure 2d), a significant reduction was found for cells grown on CNT samples with respect to the ones grown on glass, both after 12 h (from 0.71 ± 0.14 to 0.64 ± 0.14, *p* < 0.001, n = 1690 cells and n = 940 cells, respectively) and 72 h (from 0.7 ± 0.40 to 0.53 ± 0.12, *p* < 0.001, n = 2697 cells and n = 2452 cells, respectively). DOC reduction is a clear indication of substantial changes occurring in the cell morphology.

To further investigate this scenario, we then evaluated in the two cell populations the level of cell branching after 12 and 72 h on both substrates (Figure 2e, see Section 2). In both cases, there was a significant reduction in cell branching when cells were grown on CNTs with respect to glass (after 12 h: from 4.0 ± 2.3 to 1.0 ± 1.2 cusps per cell, *p* < 0.001; n = 76 cells for controls and n = 75 cells for CNTs, respectively, from 3 independent experiments; after 72 h: from 7.0 ± 5.0 to 2.9 ± 1.4 cusps per cell, *p* < 0.001; n = 409 cells for controls and n = 404 cells for CNTs, respectively, from 3 independent experiments).

Cell branching reduction, together with smaller DOC values, support the idea that CNTs promote the growth of a larger percentage of spindle-shaped/elongated cell morphologies usually associated with smooth muscle cells and fibroblasts. Remarkably, this effect does not seem to be time dependent (at least in the considered time windows).

The observed reduction in the myofibroblast-associated cell morphology in favor of SMc and Fib ones occurring in CNT-interfaced cells (about an 80% myFib reduction, Figure 2b) was then further analyzed. In particular, we marked alpha-smooth muscle actin (α-SMA), an actin isoform present in the filaments of smooth muscle cells and myofibroblasts, but not in fibroblasts [54]. An increased α-SMA expression is a well-accepted indicator of fibroblast to myofibroblast phenotypical shift [46]. We stained cells against smooth-muscle actin, using anti-α-SMA antibodies (see Section 2), on both glass controls and CNTs after 72 h (Figure 2f). We performed immunofluorescence analysis measuring α-SMA colocalization with the phalloidin-stained actin cytoskeleton (see Section 2 for details). We found that cells grown on glass substrates show a significantly higher level of α-SMA/actin colocalization when compared to the same culture developed above CNT substrates (0.83 ± 0.07 µm^2^/µm^2^ and 0.58 ± 0.09 µm^2^/µm^2^, respectively; *p* < 0.001, n = 195 cells for controls and n = 136 cells for CNTs, from three independent experiments).

The overall reduction in α-SMA cytoskeletal localization in CNT-interfaced cells (about 30% less, Figure 2g) is consistent with the observed reduction in cell morphologies associated with the SMc and myFib, α-SMA-positive, cells (25% less myFib and SMc-associated morphologies on CNTs, Figure 2b). This evidence and the remarkably high percentage of α-SMA-positive cells exclusively in SMc and myFib-classified cells (see Figure S4A) support our morphological approach in sorting pVIC populations.

### 3.4. CNTs Affect Cytoskeletal Organization, Focal Adhesions and Stiffness of VICs

**Figure 3:**
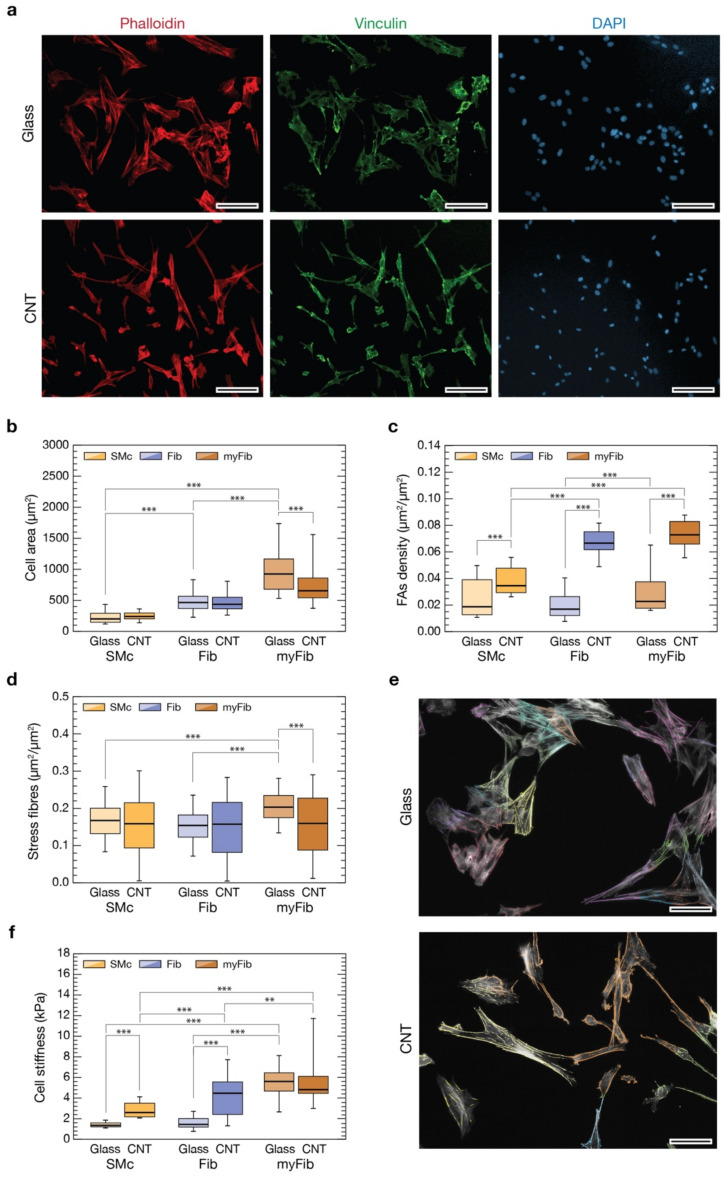
Evaluation of differences in cell parameters based on cell morphology. In (**a**) representative immunofluorescence images of cells grown on glass (top row) and CNTs (bottom row) after 72 h. Actin cytoskeleton was marked in red with phalloidin (left column), focal adhesions were highlighted in green with a staining again vinculin (middle column), and cell nuclei were made visible in blue by DAPI (right column). Scale bars: 100 µm. (**b**) Box plot summarizing cell areas distributions after 72 h from seeding on glass controls (in light orange SMc, in light blue Fib, and in light brown myFib), and on CNT substrates (in dark orange SMc, in dark blue Fib, and in dark brown myFib). (**c**) Box plot showing focal adhesion densities for the different cell morphologies when interfaced with glass and CNTs for 72 h. (**d**) Box plot summarizing the amount of stress fibers, normalized to the actin content, for the different cell morphologies when interfaced with glass and CNTs for 72 h. (**e**) Two representative fluorescent images of actin content (in light grey) of cells grown interfaced to glass controls (above) and CNTs (below) where stress fibers were highlighted for every cell with different false colors. Scale bars: 50 µm. (**f**) Box plot summarizing the cellular stiffness for the different cell morphologies when interfaced with glass and CNTs for 72 h. ** *p* < 0.01, *** *p* < 0.001.

To highlight the possible mechanism responsible for the observed shift of phenotype-associated cell morphologies, we evaluated cell cytostructural and biomechanical properties (Figure 3). Indeed, the remarkable changes in cell morphology we observed between the two substrates are likely linked to the establishment of strong cytoskeletal-driven forces used by the cell to alter its shape. The cell drives this reorganization by exerting traction forces on the substrate through anchoring points generally referred to as focal adhesions (FAs), linking the cell membrane to the substrate itself (i.e., the extracellular matrix). It is well established that their number and distribution are directly connected to the intracellular tensional state [54,55]. Moreover, the switch of fibroblast to myofibroblast is usually associated with the establishment of marked stress fibers in the actin cytoskeleton organization that, ultimately, generated from new-born or enlarged FAs [56,57].

To point out if CNT substrates have an impact on FAs, we evaluated their expression in each of the three phenotype-related cell geometries we have attempted to sort our cultures into (SMc, Fib, and myFib, on both glass and CNTs substrates). We carried out an immunofluorescence assay labelling vinculin, a cytoskeletal protein associated with the cytosolic protein complex of FAs, together with actin filaments (using phalloidin), after 72 h of growth (Figure 3a). FAs analysis was conducted evaluating the number of vinculin-positive regions normalized to the cell area for every categorized cell morphology (see Section 2). We therefore measured cell area distribution for the three distinct cell morphologies, highlighting significant differences (Figure 3b). On glass controls (lighter colors bars), SMc are the smaller ones (230 ± 102 µm^2^, n = 300 cells), myFib the larger (998 ± 408 µm^2^, n = 999 cells), while Fib fall in the between (480 ± 194 µm^2^, n = 491 cells, *p* < 0.001; data from three independent experiments). This same trend is maintained on CNT substrates (darker colors bars, *p* < 0.001, n = 332 for SMc, 945 for Fib and 294 for myFib; data from three independent experiments), confirming once more the consistency of a morphology-based cell categorization. A significant reduction in the average cell surface is appreciable only in myFib on CNTs (757 ± 326 µm^2^, *p* < 0.001) compared to controls. From these data, we can conclude that the general reduction in cell areas observed in Figure 2c might be attributable to a decreased number of myFib cells and/or to their average surface area. Both contributions point to a control effect that CNTs seem to be specifically playing on myFib development.

Previous areal analysis was functional to the evaluation of FAs densities for every cell morphology (Figure 3c). On glass controls, the actin-normalized number of FAs of Fib and SMc is similar (0.020 ± 0.012 µm^2^/µm^2^, n = 491 and 0.024 ± 0.014 µm^2^/µm^2^, n = 300, respectively), but there is a significant (50%) increase in the case of myFib (0.030 ± 0.017 µm^2^/µm^2^, *p* < 0.001, n = 999 cells). Interestingly, for every condition, FA density increased when interfaced to nanostructured CNT surfaces: a two-fold increase of FAs in SMc (0.038 ± 0.010 µm^2^/µm^2^, *p* < 0.001, n = 332 cells), and almost three-fold in Fib and myFib (0.067 ± 0.010 µm^2^/µm^2^, *p* < 0.001, n = 945 and 0.073 ± 0.011 µm^2^/µm^2^, *p* < 0.001, n = 294, respectively). This result indicates that cells have a stronger propensity to form FAs when interfaced with CNTs, and in the case of Fib and myFib cell morphologies, this effect is enhanced. Yet, this effect might correlate to an increased number or dimension of FAs (or a combination of the two).

Surprisingly, the larger number of FA-positive regions characterizing CNT-interfaced cells does not reflect a comparable increase of actin-associated stress fibers (Figure 3d,e). On glass controls, myFib-associated stress fibers are significantly more than in SMc and Fib (0.21 ± 0.05 µm^2^/µm^2^, vs. 0.16 ± 0.05 µm^2^/µm^2^ and 0.15 ± 0.05 µm^2^/µm^2^, respectively, *p* < 0.001 in both cases; n = 237 for SMc, 306 for Fib and 234 for myFib). On CNTs instead, all the cell subpopulations exhibit almost the same stress fiber density (0.17 ± 0.09 µm^2^/µm^2^, n= 206, for myFib; 0.15 ± 0.09 µm^2^/µm^2^, n = 233, for SMc; 0.16 ± 0.10 µm^2^/µm^2^, n = 254, for Fib), comparable with the ones of SMc and Fib on glass control. Despite the fact that CNTs were seemingly able to facilitate the assembling of focal adhesions in interfaced cells, such overexpression was not associated with a similar rise in stress fiber content (e.g., in the case of Fib). Moreover, in myFib cells, stress fibers were significantly less (about 20%) when interfaced on CNTs than on glass controls.

Because FAs represent the triggering units of substrate-induced cell mechanobiology [55], while stress fibers are the ultimate result of a mechano-related cytoskeleton reorganization, their alterations are possible indicators that a change in cell mechanical properties has taken place [58,59]. To evaluate this scenario, we measured cell stiffnesses by means of Atomic Force Microscopy (AFM) cell-indentation experiments [60].

AFM force spectroscopy experiments on cells cultured on glass and CNTs were performed on specific cell morphology targets by means of optical microscopy after having made cells’ cytoskeletons and nuclei visible by immunofluorescence (see Section 2). The optical transparency of our nanostructured substrates (given a CNT carpet thickness smaller than 1 µm) has made it possible to precisely position the AFM probe above each cell with high accuracy and, simultaneously, associate to every cell a specific cell morphology.

All stiffness measurements are summarized in Figure 3f. Focusing on the control glass substrate (lighter colors boxes), we found that SMc and Fib are the most compliant cells with similar stiffness (1.4 ± 0.3 kPa in Young’s Modulus value, n = 66 cells; 1.6 ± 0.7 kPa, n = 70 cells, respectively), while myFib are more than three times stiffer (about 5.5 ± 1.7 kPa, n = 147 cells, *p* < 0.001). Moving to CNT (darker colors boxes), we observed a significant increase in cell stiffness in both SMc (2.80 ± 0.80 kPa, n = 119 cells, *p* < 0.001) and Fib (4.2 ± 1.9 kPa, n = 132 cells, *p* < 0.001) when compared to controls. MyFib cells are instead as stiff as on glass (5.7 ± 2.2 kPa, n = 32 cells, *p* < 0.01), although stiffer than Fib. Overall, our data suggest that the increase in cell stiffness correlates with a larger expression of FAs when stress fibers are present, mediating pVIC mechanoadaptation.

We can speculate that CNT carpets promote the establishment of FAs in all cells but, for an unclear reason, these FAs are less prone to inducing the formation of stress fibers in myFib. Indeed, the resulting tensional state establishing within Fib cells could be insufficient to induce myFib differentiation. The mechanism responsible for this behavior seems ultimately regulated by phenomena taking place at the membrane/substrate interface; consequently, a deeper investigation of this elusive region was necessary.

### 3.5. Cell Membrane Interaction with CNTs

The ability of CNTs to regulate cell properties and behavior is connected to the direct contact taking place between CNTs and the cell membrane. Previous works have already hypothesized that CNT substrates may pierce the plasma membrane of neuronal cells plated above them, strongly affecting cell behavior [20,45,58].

**Figure 4:**
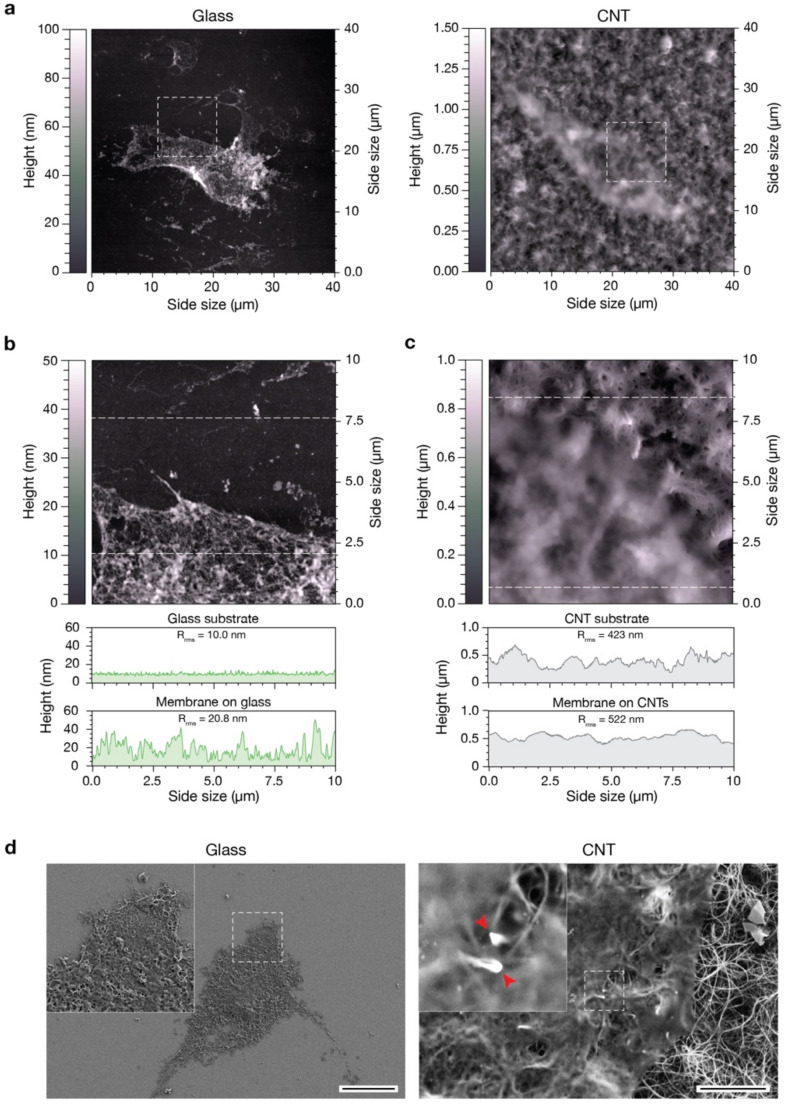
Characterization of the interaction between substrates and the inferior portion of the cell membrane. (**a**) AFM images of pVICs plasma membrane patches adherent to a flat glass substrate (left) and to a CNT carpet (right). (**b**) AFM image of the membrane lying on glass and corresponding to the region highlighted in the left image in (**a**). The two topographic profiles below correspond to a line scan on the uncovered glass substrate (top profile) and on the membrane patch (bottom profile). (**c**) AFM image of the membrane lying on CNTs and corresponding to the region highlighted in the right image in (**a**). The two topographic profiles below correspond to a line scan on the CNT carpet (top profile) and on the membrane patch (bottom profile). (**d**) Scanning electron micrographs of pVICs membrane patches adherent to a flat glass substrate (left) and to a CNT carpet (right). The two red arrows in the high magnification inset of the right image feature two carbon nanotubes interacting with the plasma membrane. Scale bars: 20 µm and 2 µm, respectively.

In order to evaluate the interaction between our CNT mats and pVIC cells, we performed a preliminary study to visualize the internal leaflet of the pVICs’ plasma membrane when grown on bare glass substrates or above CNTs. The cytosolic side of cell membranes had been exposed, breaking the cells by osmotic shock [30], and was subsequently characterized in a liquid environment through AFM imaging (Figure 4). To facilitate the optical identification of cell membrane patches, the plasma membrane was highlighted using the lipophilic membrane stain DiI (data not shown, refer to the Section 2 for details).

We analyzed cell membranes after cell lysis and washing, focusing on both membrane and substrate characteristics (Figure 4a, left and right, respectively). To obtain finer details, we acquired higher resolution images of the highlighted square portions, containing either a portion of the plasma membrane and a portion of the clean, uncovered substrate (Figure 4b,c). We compared a representative height profile of the plasma membrane and one of the uncovered glass substrate (Figure 4b, the two line-profiles below the image). The bare glass surface is flat and smooth and characterized by a linear root mean square roughness (R_rms_) of about 10 nm (top profile). As is easily predictable, the plasma membrane patch lying on such substrate is relatively flat, without any appreciable protrusion through it (R_rms_ = 20.8 nm, bottom profile). The slight increase in roughness observable is typical of this kind of preparation and is associated with the presence of intracellular protein complexes and cytoskeletal components still attached to the cytosolic side of the plasma membrane. On the other hand, the CNT carpet is characterized by a higher surface roughness (R_rms_ = 423 nm, top underneath profile in Figure 4c), mainly due to its intrinsic three-dimensional, fibrous, morphology (see Figure 1a and Figure S2) [23,24]. The membranous patch adheres to the CNT carpet following its morphology, as pointed out by the very similar surface roughness (R_rms_ = 522 nm, bottom profile).

In order to highlight possible membrane interactions with the underneath nanotubes, we prepared the same samples for an electron microscopy investigation (see Section 2). Figure 4d shows SEM images of pVIC plasma membrane patches stuck on glass (left) and CNTs (right). From a qualitative point of view, the data are well in agreement with our previous AFM results, but, interestingly, in the high magnification inset in Figure 4d, right, it is clearly visible how the CNTs below the plasma membrane are intimately in contact with it (red arrows) and apparently no ECM components interpose in the between. Therefore, we can speculate that the direct interaction taking place between CNTs and the cell membrane might not only stabilize the bilayer [45] but somehow could promote the formation and/or clustering of focal adhesion points in pVICs grown directly interfaced to them.

## 4. Discussion

Herein, we decorated fused silica surfaces with a thin layer of carbon nanotubes directly grown on the supporting substrates by CCVD, and successfully interfaced them with primary pVICs. Distinctively, substrate transparency allowed performing a multi-technique approach, combining immunofluorescence and scanning probe microscopy on cells developed above our CNT carpets.

Above nanostructured substrates, cells developed similarly to flat-glass controls (Figure 1), confirming their remarkable cytological compatibility when surface immobilized. Indeed, despite evident reductions in overall cell dimension, circularity, and branching (Figure 2), cellular densities above CNT and glass substrates are perfectly equivalent at the two time points considered in the study (12 and 72 h).

We set a cell classifier based on cell elongation of the three distinct cell morphologies characterizing our cultured pVICs: a markedly bipolar spindle-like cell shape, a spread stellate shape, and intermediate geometries ranging from unciform to sagittate shapes. These morphologies were associated with smooth muscle cells, myofibroblasts, and fibroblasts, respectively. Our analysis revealed that on CNTs the percentage of myofibroblast-like cell shapes is reduced by 80% in favor of smooth muscle and fibroblast-associated cell geometries (Figure 2). The overall significant reduction in α-SMA signal in CNT-interfaced cells supports our general hypothesis that our carbon nanostructures are able to stabilize the fibroblast-associated cell morphology, interfering with its pro-pathological myofibroblast evolution. It is worth noting that the amount of myofibroblasts characterizing our glass controls is similar to the percentage characterizing VICs from CAVD-affected valves, while, when interfaced with CNTs, the amount is closer to the percentage of healthy valves.

We extended our investigation, evaluating different cell parameters for every cell morphology. We discovered that the broad reduction in cell areas we observed is associable with a reduction in the number and/or dimension of myFib cells (Figure 3). Moreover, although CNTs seem able to facilitate the assembling of focal adhesions in interfaced cells, and in particular in Fib and myFib, this is not associated with a corresponding rise in stress fiber content. Remarkably, CNTs seem able to effectively limit the development of stress fibers, present instead in myFib on glass. Presumably, in myFib on CNTs, the larger number of FAs gives rise to a more diffuse organization of the actin fibers damping the formation of massive stress fiber bundles thanks to a more distributed transmission of load from the nanostructured substrate to the cell. Indeed, our data suggest that an increase in cell stiffness is associated with a larger expression of FAs, but this mechanoadaptation appears to be mediated by the physiological presence of stress fibers (Figure 3).

Despite the fact that our CNT substrates induce a massive over-presence of FAs, the increase in cell stiffness is appreciable only in SMc and Fib, but it was absent in the case of myFib. Apparently, it seemed that a steady-state limit was already reached in the case of Fib, and myFib could not overcome it. The opposite adaptation of Fib and myFib cell groups in terms of variation in their mechanical properties when interfaced with CNTs is partially confirmed by the literature from which these two cell phenotypes result as being differently influenced by ECM variations [7].

We attempted to investigate the role of ECM, performing AFM and SEM analysis of plasma membrane fragments adhering to both substrates. Apparently, no ECM matrix accumulates between the cells’ plasma membrane and CNTs, giving rise to a strong interlock among them (Figure 4). Therefore, we can speculate that CNTs not only stabilize the bilayer [45] but, through a not-yet-clarified mechanism, promote the formation and/or clustering of FAs in pVICs.

## 5. Conclusions

This study demonstrated the possibility of decorating material surfaces with strongly adherent CNTs. This makes it possible to design biodevices endowed with nano-morphological cues able to modulate cellular adaptation and evolution for tissue engineering applications [61].

The results obtained indicate that the (nano)-morphology of CNTs greatly affects the behavior of interfaced pVICs. In particular, CNTs have been demonstrated to have a positive effect in settling pVIC entanglement into a morphology attributable to the fibroblast phenotype instead of myofibroblasts, keeping them in a sort of “quiescent state” [62]. This effect could be reconnected to a better redistribution of the tensional stress within cells, avoiding exceeding the activation limit inducing Fib-to-myFib differentiation. In the context of CAVD, our discovery shed some light on the impact that connective and supporting tissues nanomorphology could have on assuring parenchymal tensional homeostasis, preventing the pro-pathological evolution of the cell linage. This discovery potentially opens the door to a new comprehension of the relationship between ECM morphology and the onset of a pro-pathological evolution in the different valve-resident cells [63].

Our CNT substrates, with their peculiar morphological similarity to the cellular ECM, impacted cell mechanoadaptation, stabilizing a fibroblast-associated cell morphology. However, account must be taken of the fact that our investigation was mainly phenomenological. The phenotypical heterogeneity of VICs that we attempted to recap based on a morphological classification needs to be assessed through a complemental functional genomic investigation or proteomic screening. RNA sequencing, quantitative PCR or flow cytometry analysis may highlight gene expression changes and/or altered protein levels in cells interfaced to our two substrates, substantiating the genetic fingerprint and adaptation of VICs interfaced with CNTs.

Although more fundamental research is necessary, our discovery opens the door to new approaches for tissue engineering applications where (nano)morphological cues could enhance cell interfacing, regulate mechanoadaptation and, potentially, drive cell phenotype evolution.

## Data Availability

The data presented in this study are available on request from the corresponding authors.

## Supplementary Materials

The following are available online at https://www.mdpi.com/article/10.3390/nano11102724/s1, Figure S1: Scanning electron micrographs of CNT carpets grown above a supporting fused silica coverslip; Figure S2: Representative examples of cell morphologies and relative elongations (Δ); Figure S3: Scanning electron micrographs of four different AFM probes; Figure S4: Evaluation of cell molecular signature and elevation based on cell phenotype-associated morphology; Figure S5: Representative fields comparing α-SMA staining of glass and CNT-interfaced cells.
