# Supplementary material for: Carbon Nanotubes Substrates Alleviate Pro-Calcific Evolution in Porcine Valve Interstitial Cells
Source: Nanomaterials (Basel). 2021 Oct 15;11(10):2724. doi: 10.3390/nano11102724 (PMC8538037; doi:10.3390/nano11102724)
Supplement: Supplementary file 1 [file nanomaterials-11-02724-s001.zip › nanomaterials-1377190-SI.pdf]

Carbon Nanotubes Substrates Alleviate Pro-Calcific Evolution in Porcine Valve Interstitial Cells

Luisa Severino Ulloa [1], Fabio Perissinotto [1], Ilaria Rago [1], Andrea Goldoni [2], Rosaria Santoro [3], Maurizio Pesce [3], Loredana Casalis [2,*] and Denis Scaini [4,5,*]

1 Dipartimento di Fisica, Università di Trieste, Piazzale Europa 1, 34127 Trieste, Italy; luisa.ulloaseverino@unityhealth.to (L.S.U.); fabio.perissinotto@inserm.fr (F.P.); ilaria.rago@uniroma1.it (I.R.)

2 Elettra-Sincrotrone Trieste S.C.p.A., Basovizza, 34149 Trieste, Italy; andrea.goldoni@elettra.eu

3 Unità di Ingegneria Tissutale Cardiovascolare, Centro Cardiologico Monzino, IRCCS, 20138 Milan, Italy; rosaria.santoro@cardiologicomonzino.it (R.S.); Maurizio.Pesce@cardiologicomonzino.it (M.P.)

4 Scuola Internazionale Superiore di Studi Avanzati, via Bonomea 265, 34136 Trieste, Italy

5 Faculty of Medicine, Imperial College London, London W12 0NN, UK

* Correspondence: loredana.casalis@elettra.eu (L.C.); d.scaini@imperial.ac.uk (D.S.)

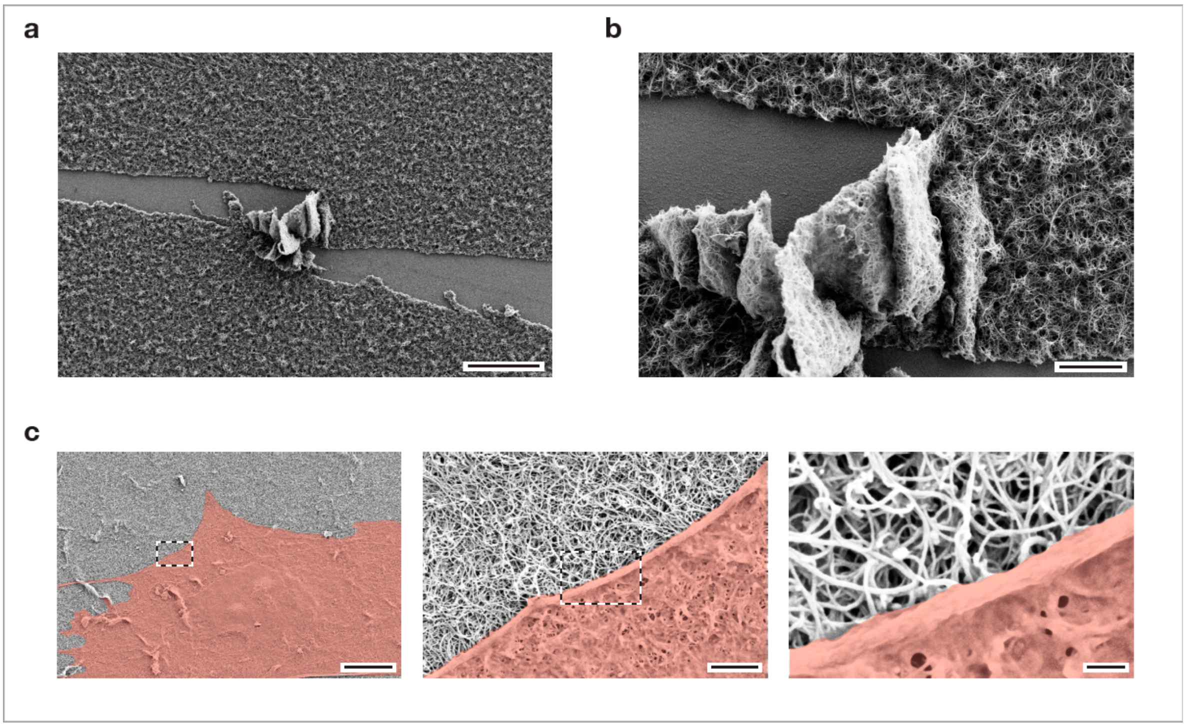

Figure S1. Scanning electron micrographs of CNT carpets grown above a supporting fused silica coverslip.

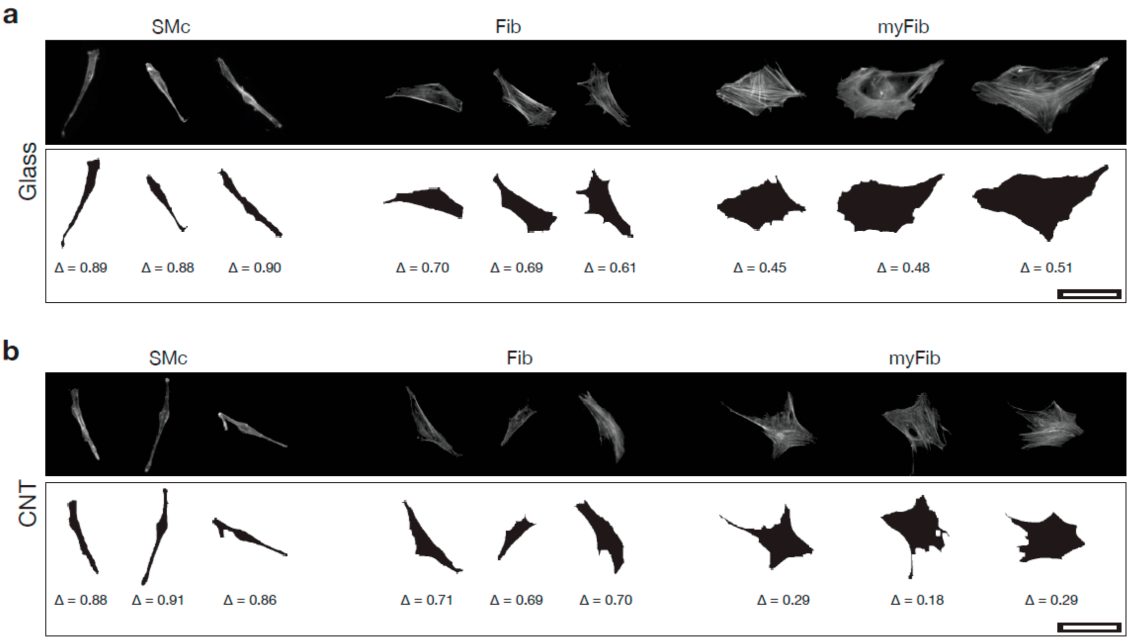

Figure S2. Representative examples of cell morphologies and relative elongations (Δ).

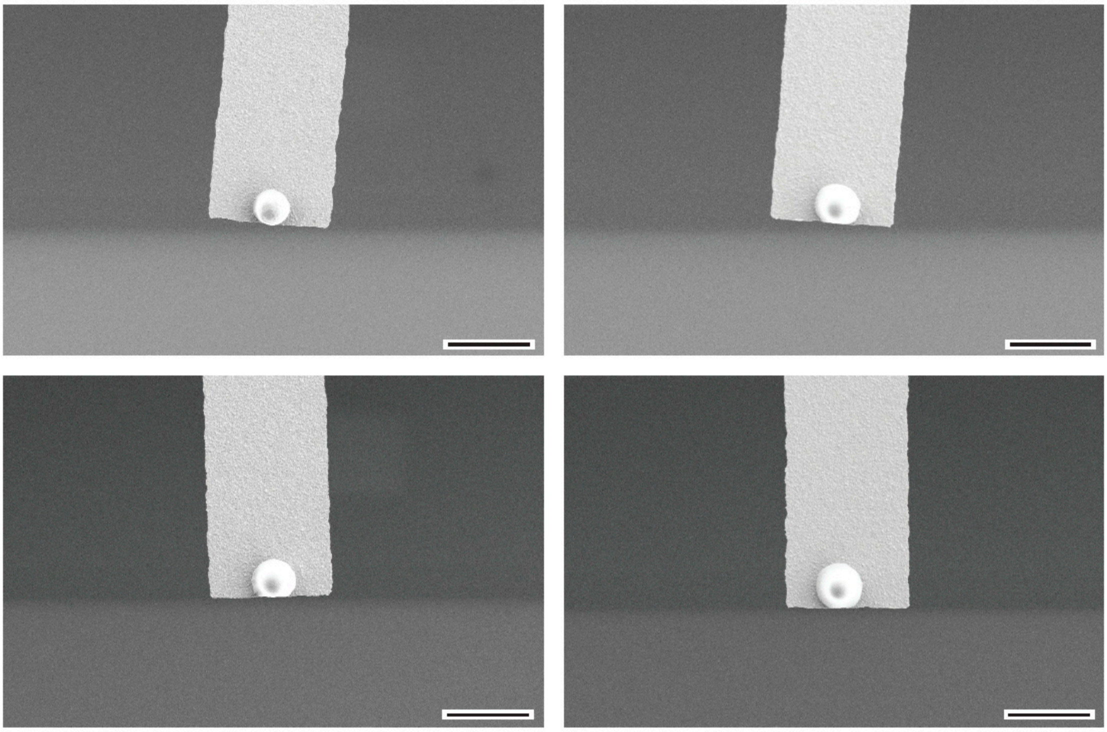

Figure S3. Scanning electron micrographs of four different AFM probes.

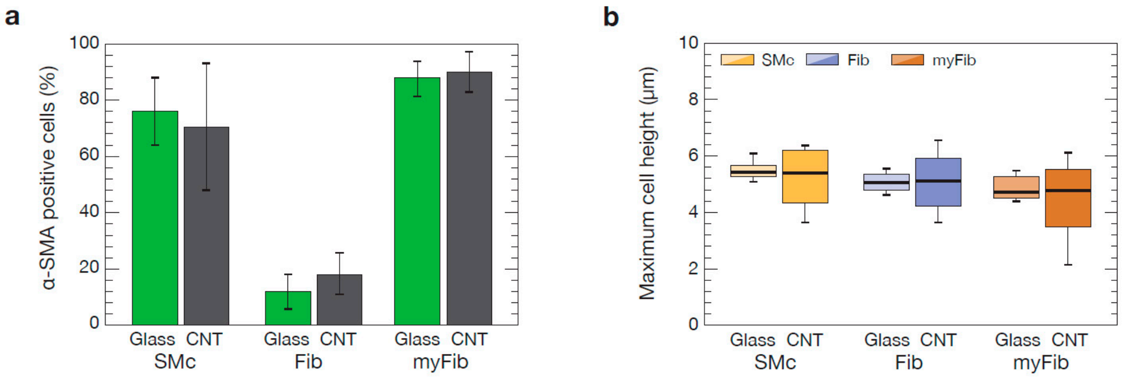

Figure S4. Evaluation of cell molecular signature and elevation based on cell phenotype-associated morphology.

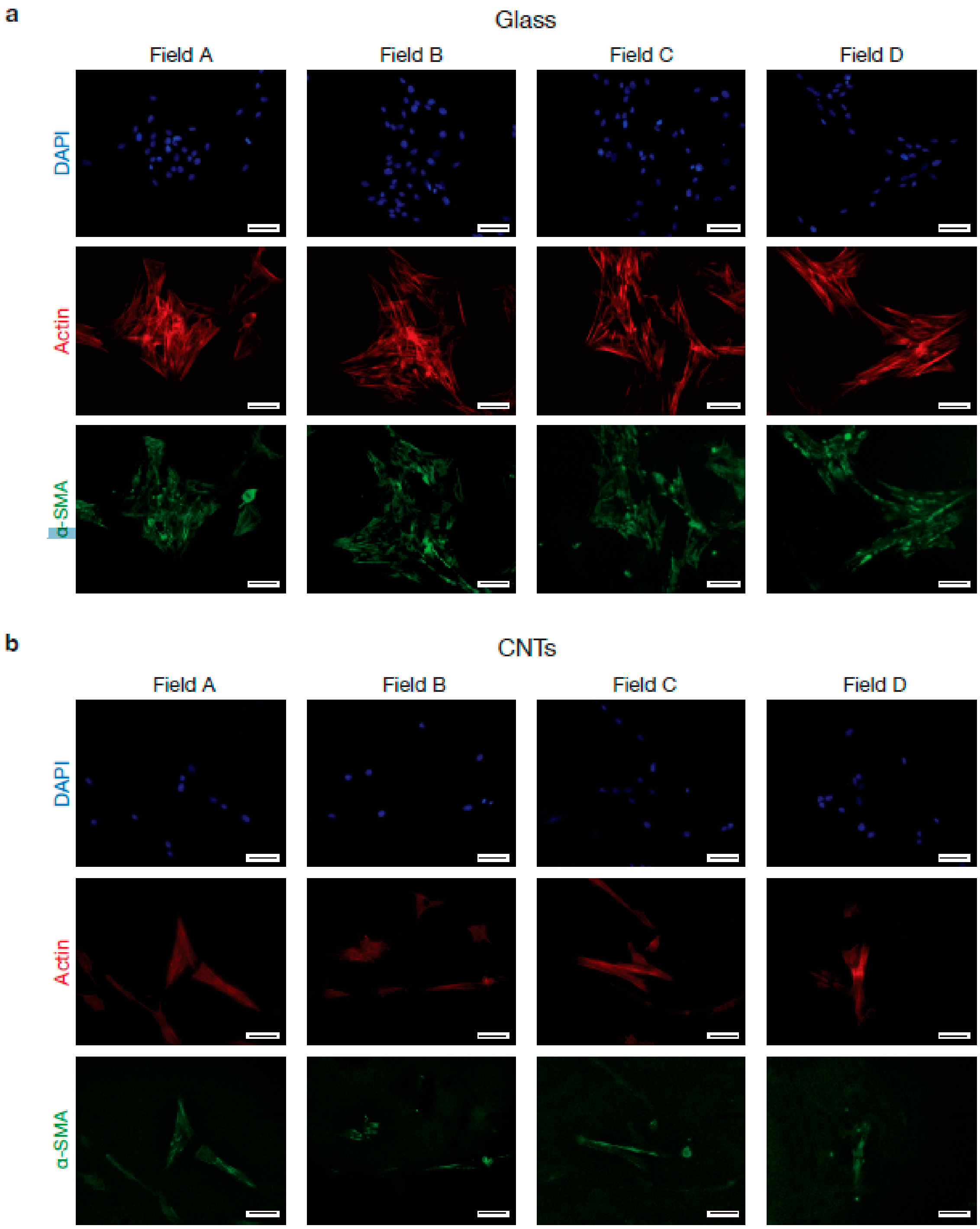

Figure S5: Representative fields comparing α-SMA staining of glass and CNT-interfaced cells.
